# Crystal structure and Hirshfeld surface analysis of 5,5-di­chloro-2-(di­chloro­meth­yl)-6,6-dimethyl-5,6-di­hydro­pyrimidin-4-amine

**DOI:** 10.1107/S2056989025009247

**Published:** 2025-11-06

**Authors:** Atash V. Gurbanov, Mehmet Akkurt, Gizachew Mulugeta Manahelohe

**Affiliations:** a Excellence Center, Baku State University, Z. Khalilov Str. 33, AZ 1148, Baku, Azerbaijan; b Department of Physics, Faculty of Sciences, Erciyes University, 38039 Kayseri, Türkiye; c Department of Chemistry, University of Gondar, PO Box 196, Gondar, Ethiopia; Katholieke Universiteit Leuven, Belgium

**Keywords:** crystal structure, 4,5-di­hydro­pyrimidine, hydrogen bonds, dimers, Hirshfeld surface analysis

## Abstract

In the title compound, C_7_H_9_Cl_4_N_3_, the central 4,5-di­hydro­pyrimidine ring adopts an approximate twist-boat conformation. In the crystal, mol­ecules are connected in the [101] direction by ribbons of N—H⋯N hydrogen-bonded dimers with an *R*^2^_2_(8) motif.

## Abstract 2

In the crystal, N—H⋯N hydrogen bonds of *R*^2^_2_(8) motif dimers generate ribbons of mol­ecules in the crystal that are linked in the [101] direction.

## 1. Chemical context

N-containing compounds have attracted much attention due to their properties in the fields of mol­ecular recognition, crystal engineering, catalysis, coordination chemistry and organic synthesis (Gadzhieva *et al.*, 2005 ▸; Maharramov *et al.*, 2011 ▸; Gurbanov *et al.*, 2022 ▸; Polyanskii *et al.*, 2019 ▸). Depending on the main N-skeleton as well as the attached substituents, the supra­molecular arrangements and catalytic activity of the corresponding metal complexes can be regulated (Aliyeva *et al.*, 2024 ▸; Gurbanov *et al.*, 2018 ▸; Huseynov *et al.*, 2018 ▸). Numerous synthetic strategies for the synthesis of new N-containing compounds have been developed including metal-mediated synthesis (Gurbanov *et al.*, 2023 ▸; Mahmudov *et al.*, 2023 ▸). Attachment of –Cl and –NH_2_ groups on the N-heterocycle can alter the supra­molecular mode of the corresponding organic materials. Thus, in the current work we have synthesized the title compound, which provides multiple inter­molecular non-covalent inter­actions.
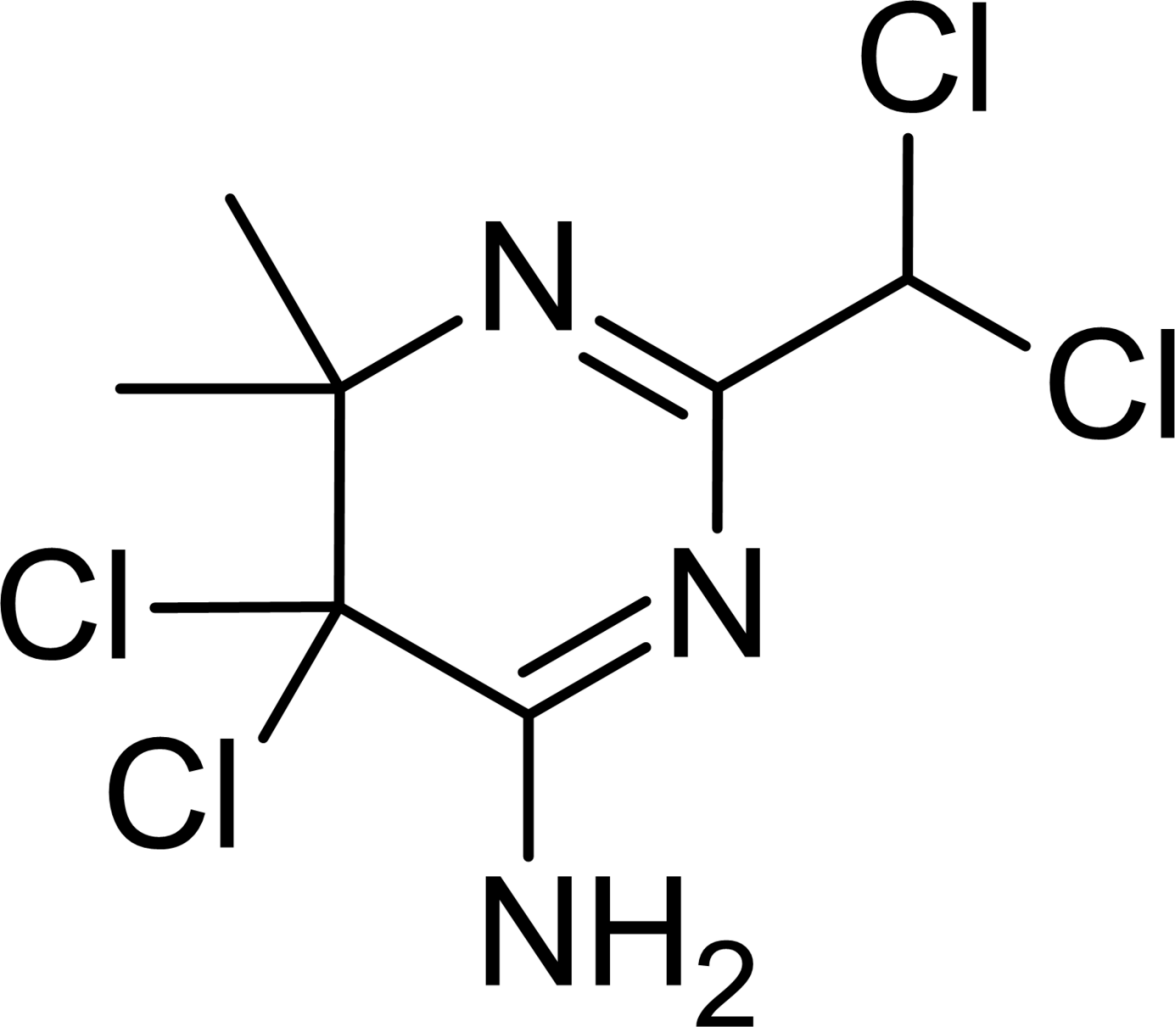


## 2. Structural commentary

**Figure 1:**
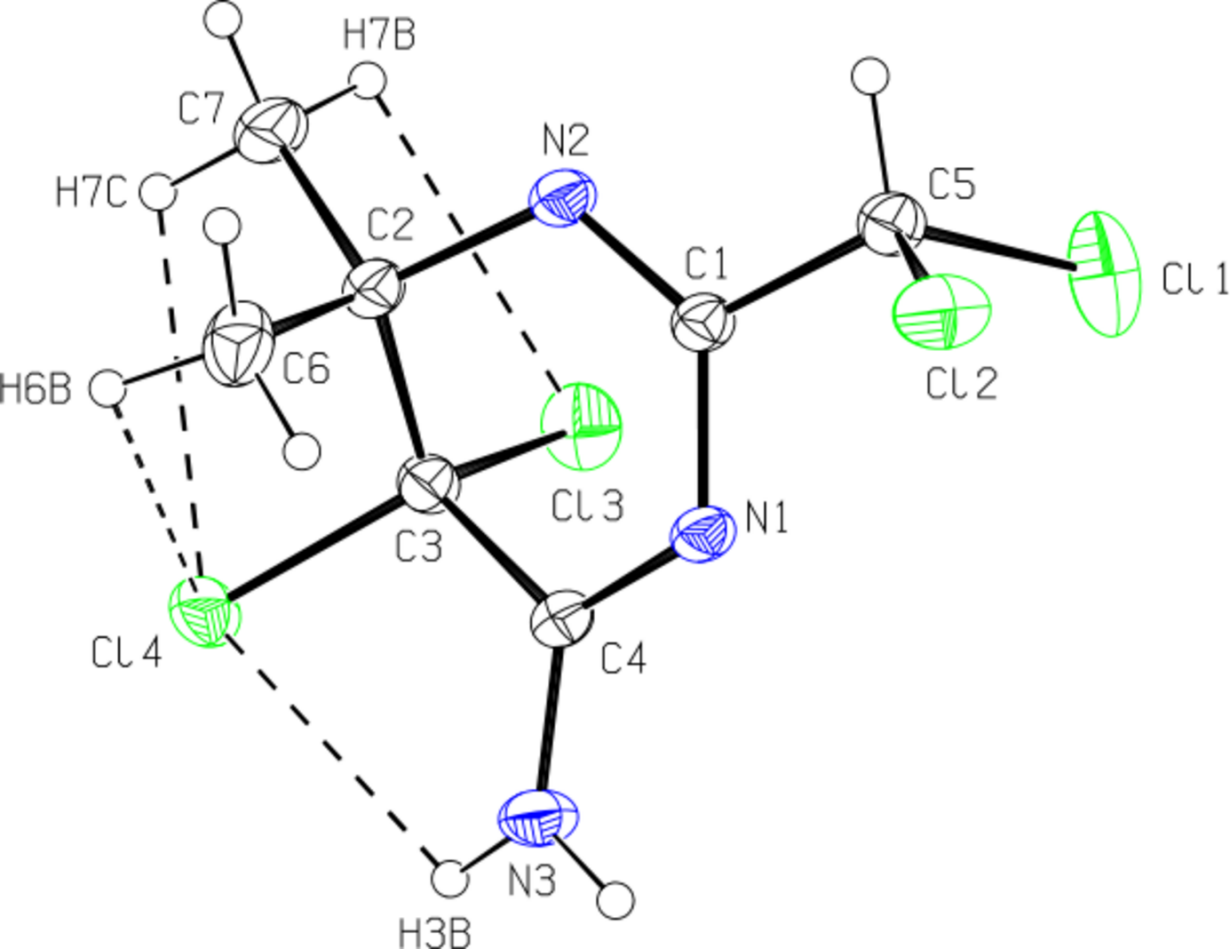
The title mol­ecule with the atom-labelling scheme and displacement ellipsoids drawn at the 30% probability level. Short intramolecular H⋯Cl contacts are indicated by dashed lines.

The central 4,5-di­hydro­pyrimidine ring exhibits an approximate twist-boat conformation. Atoms C1/C2/N1/N2 are almost coplanar (r.m.s. deviation = 0.006 Å) and C3 and C4 deviate from their best plane by 0.824 (2) and 0.317 (2) Å, respectively. The molecule features a short intramolecular N—H⋯Cl contact (Fig. 1 ▸; Table 1 ▸) forming a *C*(5) motif (Bernstein *et al.*, 1995 ▸). The C—N distances in the 4,5-di­hydro­pyrimidine ring are consistent with single- and double-bond characteristics [C1=N2 = 1.276 (2), C4=N1 = 1.307 (2) C2—N2 = 1.473 (2), C1—N1 = 1.387 (2) and C4—N3 = 1.313 (2) Å]. The single C—N bond length [1.313 (2) Å] for the NH_2_ group attached to the pyrimidine ring is significantly shorter than a typical C—N single bond (around 1.47 Å). The other bond lengths and angles are comparable to those in the structures discussed in the *Database survey* section.

## 3. Supra­molecular features and Hirshfeld surface analysis

**Figure 2:**
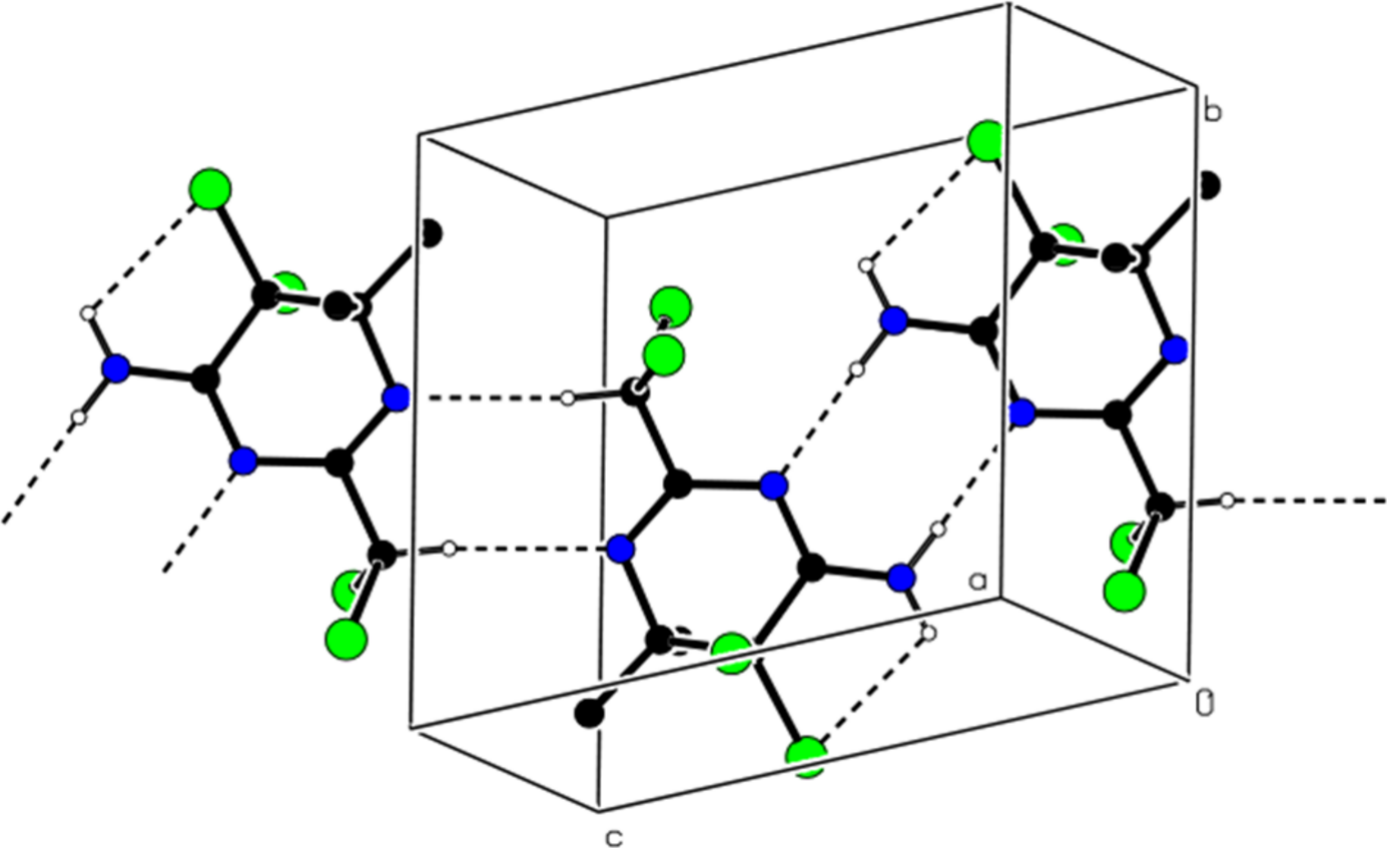
Partial packing of the title compound in the unit cell, showing N—H⋯N and C—H⋯N hydrogen bonds as dashed lines. H atoms not involved in hydrogen bonding have been omitted for clarity.

In the crystal, mol­ecules are linked into dimers with an 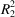
(8) motif by N—H⋯N hydrogen bonds, forming ribbons in the [10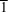] direction, which also feature pairwise C—H⋯N hydrogen bonds (Table 1 ▸; Figs. 2 ▸, 3 ▸ and 4 ▸). No C—H⋯π or π–π inter­actions are found. van der Waals inter­actions between the ribbons consolidate the crystal structure.

**Figure 5:**
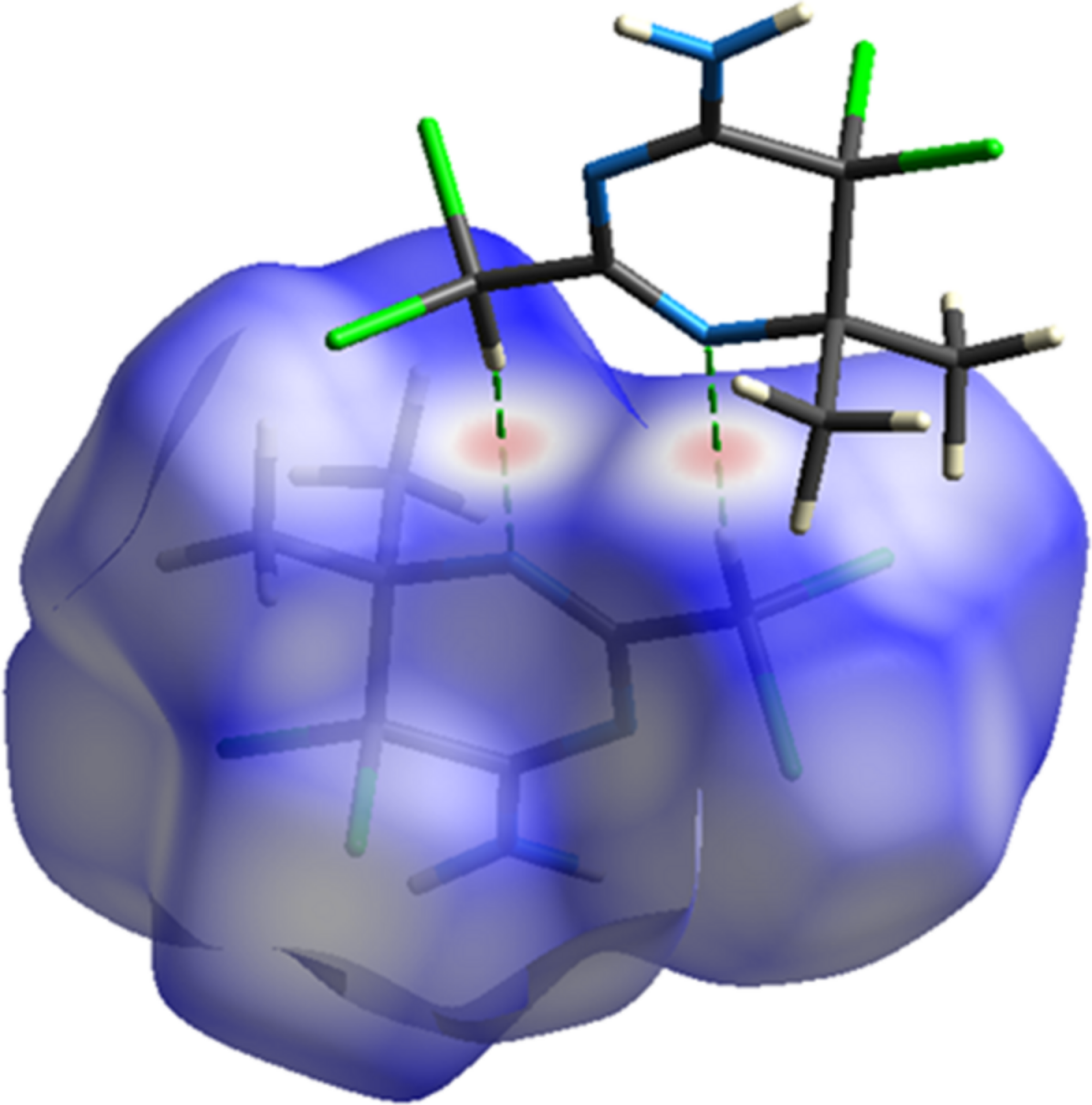
View of the three-dimensional Hirshfeld surface of the compound plotted over *d*_norm_.

**Figure 6:**
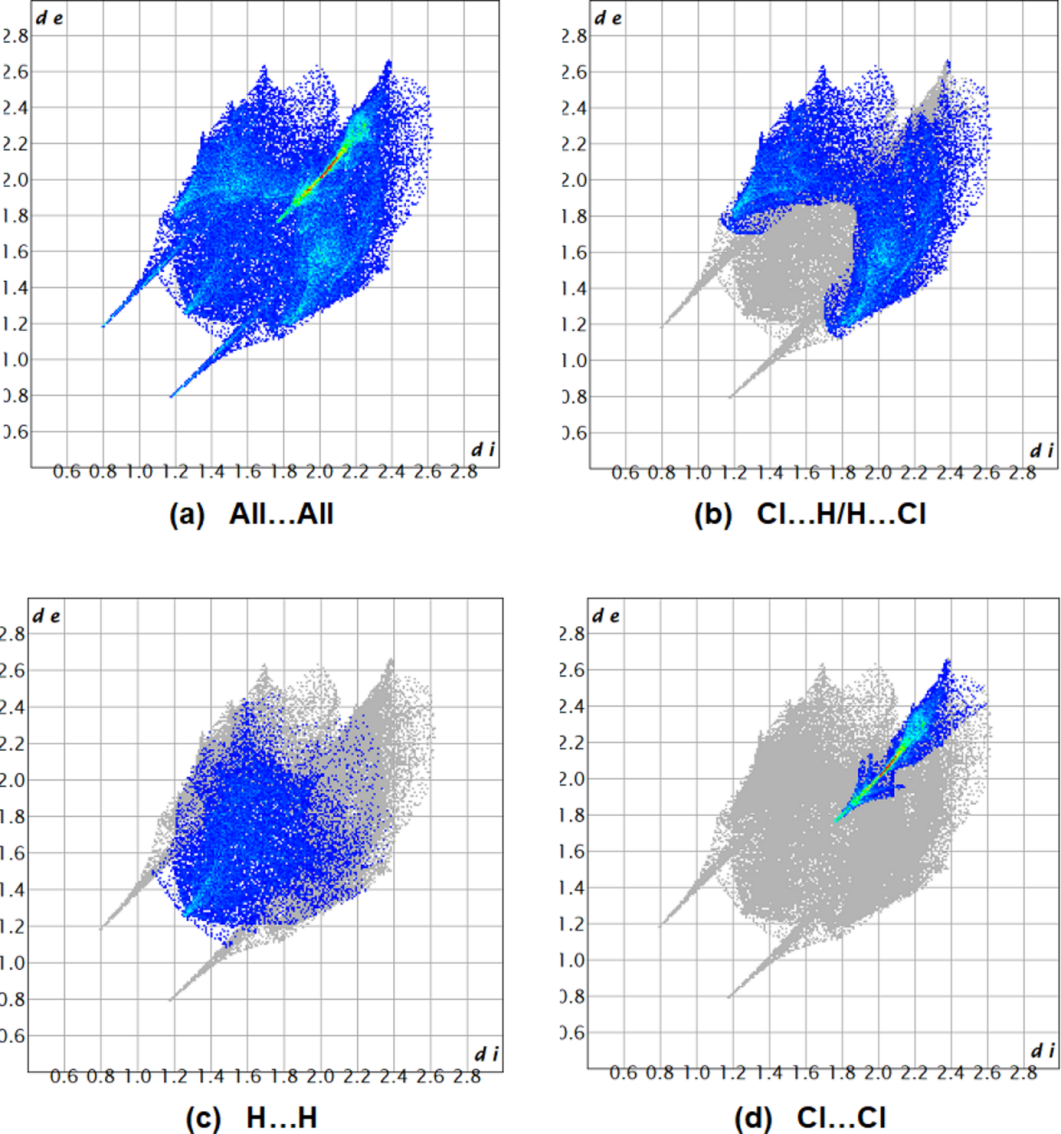
The two-dimensional fingerprint plots, showing (*a*) all inter­actions, and delineated into (*b*) Cl⋯H/H⋯Cl, (*c*) H⋯H and (*d*) Cl⋯Cl inter­actions [*d*_e_ and *d*_i_ represent the distances from a point on the Hirshfeld surface to the nearest atoms outside (external) and inside (inter­nal) the surface, respectively].

*Crystal Explorer* 17.5 (Spackman *et al.*, 2021 ▸) was utilized to generate Hirshfeld surfaces (Fig. 5 ▸) and two-dimensional fingerprint plots (Fig. 6 ▸) in order to qu­antify the inter­molecular inter­actions in the crystal (Table 1 ▸). The most important Cl⋯H/H⋯Cl inter­actions appear as two symmetrical broad wings with *d*_e_ + *d*_i_ = 2.85 Å and contribute 42.9% to the Hirshfeld surface (Fig. 6 ▸*b*). The inter­molecular H⋯H contacts, contributing 25.9% to the overall crystal packing, are reflected in Fig. 6 ▸*c* as widely scattered points of high density due to the large hydrogen content of the mol­ecule, with the tip at *d*_e_ = *d*_i_ = 1.25 Å. The observed Cl⋯Cl contact distance of 3.5355 (8) Å is slightly longer than the conventional 3.50 Å van der Waals separation. The Cl⋯Cl contacts (16.2%) have an arrow-shaped distribution of points with the tip at *d*_e_ = *d*_i_ = 1.75 Å (Fig. 6 ▸*d*). The N⋯H/H⋯N inter­actions represent 9.5% of the total Hirshfeld surface. These inter­actions are manifested as two symmetrical sharp spikes at *d*_e_ + *d*_i_ = 1.95 Å (Fig. 6 ▸*a*). The Cl⋯N/N⋯Cl (4.4%), Cl⋯C/C⋯Cl (0.7%) and C⋯H/H⋯C (0.4%) inter­actions all contribute in smaller ways.

## 4. Database survey

A search of the Cambridge Structural Database (CSD, Version 6.00, update of April 2025; Groom *et al.*, 2016 ▸) for 4,5-di­hydro­pyrimidine resulted in 77 hits. Entries KIMHIB (Wan *et al.*, 2023 ▸) and ZEDLOJ (Mori & Maeda, 1994 ▸) are the closest analogues of the title compound.

KIMHIB crystallizes with two independent mol­ecules (*A* and *B*) in the asymmetric unit of the ortho­rhom­bic space group *Pna*2_1_. ZEDLOJ crystallizes in the monoclinic space group *A*2/*a*. In KIMHIB, the di­hydro­pyrimidine ring of mol­ecule *A* adopts a distorted screw-boat conformation with ring puckering parameters *Q_T_* = 0.433 (6) Å, θ = 112.2 (8)° and φ = 328.7 (9)°, whereas the di­hydro­pyrimidine ring of mol­ecule *B* exhibits a distorted twist-boat conformation [*Q*_T_ = 0.459 (6) Å, θ = 109.2 (7)°, φ = 81.1 (8)°]. In ZEDLOJ, the central pyrimidine ring adopts a distorted screw-boat conformation [*Q*_T_ = 0.462 (2) Å, θ = 67.2 (2) °, φ = 216.1 (3) °]. Idealized values for screw-boat and twist-boat conformations are: θ = 67.5° and 90°, and φ = (60*k* + 30)°, respectively, where *k* is an integer.

In KIMHIB, the mol­ecular conformation may be associated with C—H⋯N intra­molecular inter­actions. In the crystal, mol­ecules form layers parallel to the (100) plane *via* C—H⋯π inter­actions with van der Waals inter­actions between the layers, no π–π inter­actions are observed. In ZEDLOJ, the mol­ecular conformation may be supported by C—H⋯N hydrogen bonds. Classical inter­molecular hydrogen bonds are not observed, with C—H⋯π and van der Waals inter­actions consolidating of the structure.

## 5. Synthesis and crystallization

To a mixture of 0.5 ml di­chloro­aceto­nitrile and 1.5 ml of NH_4_OH (28–30%) solution was added 10 mg of Pd(CH_3_COO)_2_ in 5 ml acetone. The mixture was stirred for 24 h at r.t. The precipitate was filtered and dissolved in CH_2_Cl_2_. Light-yellow crystals of the title compound suitable for X-ray structural analysis were obtained after *ca* 2 d. Yield 60%; IR (ATR, 298 K): 3320 and 3203 *ν*(N—H), 1641 and 1602 *ν*(C=N); *M*_r_ = 276.97; elemental analysis calculated (%) for C_7_H_9_Cl_4_N_3_: C 30.36, H 3.28, N 15.17; found: C 30.33, H 3.27, N 15.14. ^1^H NMR in DMSO*-d*_6_, δ(p.p.m.): 1.98 (3H, –CH_3_) and 2.07 (3H,–CH_3_), 5.91 (1H, –CHCl_2_), 8.35 (2H,–NH_2_). ^13^C NMR in DMSO-*d*_6_, *δ* (p.p.m.): 18.62 and 23.65 (–CH_3_), 67.02 (–CCH_3_), 70.99 (–CHCl_2_), 112.44 (–CCl_2_–), 150.51 and 165.34 (C=N).

## 6. Refinement

Crystal data, data collection and structure refinement details are summarized in Table 2 ▸. The hydrogen atoms were placed in calculated positions and refined as riding models with fixed isotropic displacement parameters [C—H = 0.96 and 0.98 Å, N—H = 0.90 Å with *U*_iso_(H) = 1.2*U*_eq_(N, C)].

## Supplementary Material

Crystal structure: contains datablock(s) I. DOI: 10.1107/S2056989025009247/vm2318sup1.cif

Structure factors: contains datablock(s) I. DOI: 10.1107/S2056989025009247/vm2318Isup3.hkl

Supporting information file. DOI: 10.1107/S2056989025009247/vm2318Isup3.cml

CCDC reference: 2237849

Additional supporting information:  crystallographic information; 3D view; checkCIF report

## Figures and Tables

**Figure 3:**
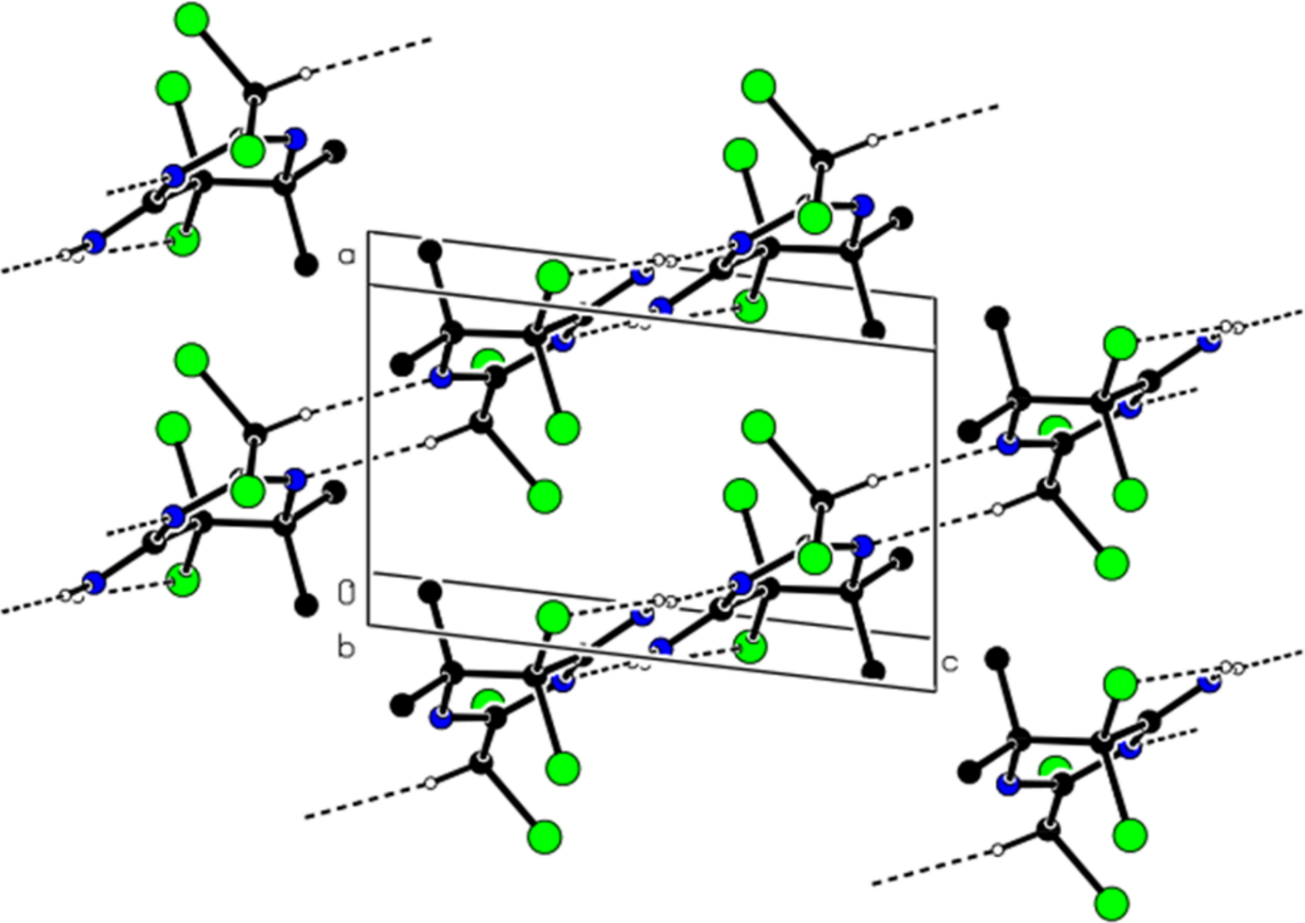
The view of the inter­actions shown in Fig. 2 ▸ from the *b-*axis.

**Figure 4:**
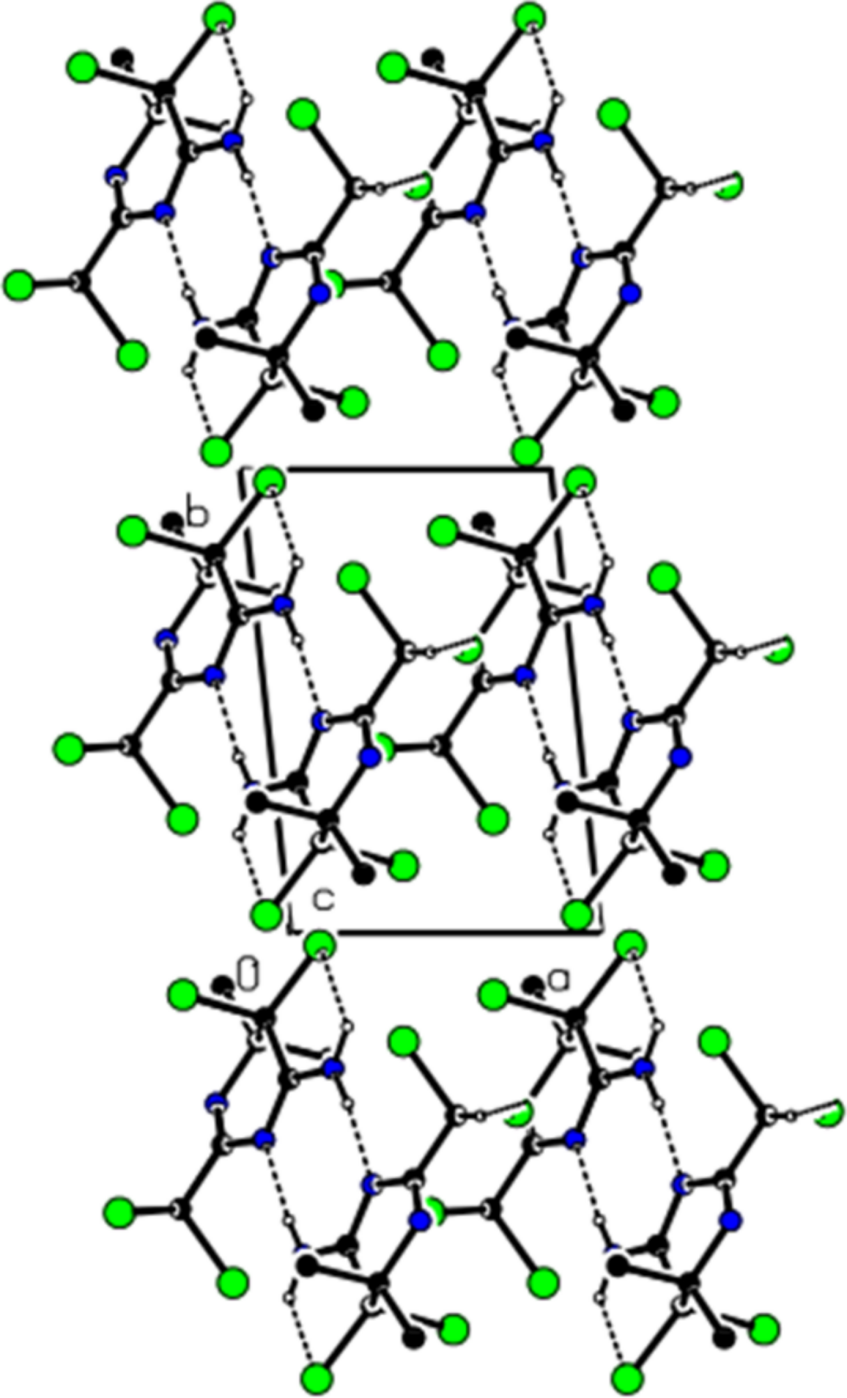
The view of the inter­actions shown in Fig. 2 ▸ from the *c*-axis.

**Table 1:** Hydrogen-bond geometry (Å, °)

*D*—H⋯*A*	*D*—H	H⋯*A*	*D*⋯*A*	*D*—H⋯*A*
N3—H3*A*⋯N1^i^	0.90	2.08	2.978 (2)	174
C5—H5*A*⋯N2^ii^	0.98	2.54	3.506 (3)	170

Symmetry codes: (i) 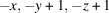; (ii) 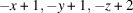.

**Table 2:** Experimental details

Crystal data	
Chemical formula	C_7_H_9_Cl_4_N_3_
*M*_r_	276.97
Crystal system, space group	Triclinic, *P* 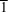
Temperature (K)	296
*a*, *b*, *c* (Å)	6.1069 (3), 9.1118 (4), 10.2591 (5)
α, β, γ (°)	90.981 (2), 96.700 (2), 95.980 (2)
*V* (Å^3^)	563.63 (5)
*Z*	2
Radiation type	Mo *K*α
μ (mm^−1^)	1.01
Crystal size (mm)	0.25 × 0.18 × 0.12

Data collection	
Diffractometer	Bruker D8 Quest PHOTON 100 detector
Absorption correction	Multi-scan (*SADABS*; Krause *et al.*, 2015 ▸
*T*_min_, *T*_max_	0.793, 0.874
No. of measured, independent and observed [*I* > 2σ(*I*)] reflections	8178, 2272, 1998
*R*_int_	0.037
(sin θ/λ)_max_ (Å^−1^)	0.626

Refinement	
*R*[*F*^2^ > 2σ(*F*^2^)], *wR*(*F*^2^), *S*	0.033, 0.083, 1.07
No. of reflections	2272
No. of parameters	129
H-atom treatment	H-atom parameters constrained
Δρ_max_, Δρ_min_ (e Å^−3^)	0.39, −0.36

Computer programs: *APEX3* and *SAINT* (Bruker, 2008 ▸), *SHELXS97* (Sheldrick, 2008 ▸), *SHELXL2014* (Sheldrick, 2015 ▸), *ORTEP-3 for Windows* (Farrugia, 2012 ▸) and *PLATON* (Spek, 2020 ▸).

## supplementary crystallographic information

### 5,5-Dichloro-2-(dichloromethyl)-6,6-dimethyl-5,6-dihydropyrimidin-4-amine Crystal data

**Table:**

C_7_H_9_Cl_4_N_3_	*Z* = 2
*M**_r_* = 276.97	*F*(000) = 280
Triclinic, *P*1	*D*_x_ = 1.632 Mg m^−^^3^
*a* = 6.1069 (3) Å	Mo *K*α radiation, λ = 0.71073 Å
*b* = 9.1118 (4) Å	Cell parameters from 5453 reflections
*c* = 10.2591 (5) Å	θ = 3.1–26.4°
α = 90.981 (2)°	µ = 1.01 mm^−^^1^
β = 96.700 (2)°	*T* = 296 K
γ = 95.980 (2)°	Plate, light-yellow
*V* = 563.63 (5) Å^3^	0.25 × 0.18 × 0.12 mm

### 5,5-Dichloro-2-(dichloromethyl)-6,6-dimethyl-5,6-dihydropyrimidin-4-amine Data collection

**Table:**

Bruker D8 Quest PHOTON 100 detector diffractometer	1998 reflections with *I* > 2σ(*I*)
Detector resolution: 0 pixels mm^-1^	*R*_int_ = 0.037
φ and ω scans	θ_max_ = 26.4°, θ_min_ = 2.3°
Absorption correction: multi-scan (SADABS; Krause *et al.*, 2015	*h* = −7→7
*T*_min_ = 0.793, *T*_max_ = 0.874	*k* = −11→11
8178 measured reflections	*l* = −12→11
2272 independent reflections	

### 5,5-Dichloro-2-(dichloromethyl)-6,6-dimethyl-5,6-dihydropyrimidin-4-amine Refinement

**Table:**

Refinement on *F*^2^	Primary atom site location: structure-invariant direct methods
Least-squares matrix: full	Secondary atom site location: structure-invariant direct methods
*R*[*F*^2^ > 2σ(*F*^2^)] = 0.033	Hydrogen site location: mixed
*wR*(*F*^2^) = 0.083	H-atom parameters constrained
*S* = 1.07	*w* = 1/[σ^2^(*F*_o_^2^) + (0.0247*P*)^2^ + 0.3903*P*] where *P* = (*F*_o_^2^ + 2*F*_c_^2^)/3
2272 reflections	(Δ/σ)_max_ = 0.001
129 parameters	Δρ_max_ = 0.39 e Å^−^^3^
0 restraints	Δρ_min_ = −0.36 e Å^−^^3^

### 5,5-Dichloro-2-(dichloromethyl)-6,6-dimethyl-5,6-dihydropyrimidin-4-amine Special details

**Table:**

Geometry. All esds (except the esd in the dihedral angle between two l.s. planes) are estimated using the full covariance matrix. The cell esds are taken into account individually in the estimation of esds in distances, angles and torsion angles; correlations between esds in cell parameters are only used when they are defined by crystal symmetry. An approximate (isotropic) treatment of cell esds is used for estimating esds involving l.s. planes.

### 5,5-Dichloro-2-(dichloromethyl)-6,6-dimethyl-5,6-dihydropyrimidin-4-amine Fractional atomic coordinates and isotropic or equivalent isotropic displacement parameters (Å^2^)

**Table:**

	*x*	*y*	*z*	*U*_iso_*/*U*_eq_	
C1	0.2993 (3)	0.46088 (19)	0.77624 (17)	0.0290 (4)	
C2	0.1477 (3)	0.2365 (2)	0.85204 (18)	0.0327 (4)	
C3	0.1199 (3)	0.18825 (19)	0.70582 (18)	0.0291 (4)	
C4	0.0636 (3)	0.3194 (2)	0.62278 (17)	0.0281 (4)	
C5	0.4578 (3)	0.5991 (2)	0.8006 (2)	0.0368 (4)	
H5A	0.535164	0.597285	0.889662	0.044*	
C6	−0.0758 (4)	0.2743 (3)	0.8909 (2)	0.0470 (5)	
H6A	−0.053847	0.318689	0.977638	0.071*	
H6B	−0.176096	0.185742	0.889584	0.071*	
H6C	−0.136976	0.342158	0.829808	0.071*	
C7	0.2421 (4)	0.1190 (2)	0.9398 (2)	0.0471 (5)	
H7A	0.238776	0.146708	1.030174	0.071*	
H7B	0.392426	0.110595	0.924333	0.071*	
H7C	0.154603	0.025863	0.920024	0.071*	
Cl1	0.65595 (12)	0.60821 (8)	0.68869 (9)	0.0749 (2)	
Cl2	0.31392 (11)	0.75861 (6)	0.78797 (6)	0.05086 (18)	
Cl3	0.37541 (9)	0.13811 (6)	0.65640 (6)	0.04623 (16)	
Cl4	−0.08182 (8)	0.03163 (5)	0.67338 (5)	0.04116 (15)	
N1	0.1654 (3)	0.45015 (16)	0.65682 (14)	0.0299 (3)	
N2	0.3036 (3)	0.37185 (17)	0.87098 (15)	0.0344 (4)	
N3	−0.0794 (3)	0.29820 (18)	0.51650 (16)	0.0399 (4)	
H3A	−0.114976	0.373990	0.465988	0.048*	
H3B	−0.140745	0.207580	0.487858	0.048*	

### 5,5-Dichloro-2-(dichloromethyl)-6,6-dimethyl-5,6-dihydropyrimidin-4-amine Atomic displacement parameters (Å^2^)

**Table:**

	*U*^11^	*U*^22^	*U*^33^	*U*^12^	*U*^13^	*U*^23^
C1	0.0314 (9)	0.0258 (8)	0.0287 (9)	0.0033 (7)	−0.0013 (7)	0.0040 (7)
C2	0.0379 (10)	0.0298 (9)	0.0281 (9)	−0.0019 (8)	−0.0017 (7)	0.0068 (7)
C3	0.0288 (8)	0.0252 (8)	0.0320 (9)	0.0023 (7)	−0.0016 (7)	0.0036 (7)
C4	0.0304 (9)	0.0296 (9)	0.0243 (8)	0.0051 (7)	0.0014 (7)	0.0044 (7)
C5	0.0409 (10)	0.0280 (9)	0.0384 (10)	−0.0005 (8)	−0.0062 (8)	0.0081 (8)
C6	0.0519 (13)	0.0490 (13)	0.0421 (12)	0.0011 (10)	0.0176 (10)	0.0011 (10)
C7	0.0578 (13)	0.0369 (11)	0.0411 (12)	−0.0043 (10)	−0.0108 (10)	0.0179 (9)
Cl1	0.0567 (4)	0.0623 (4)	0.1098 (6)	−0.0065 (3)	0.0378 (4)	0.0052 (4)
Cl2	0.0716 (4)	0.0310 (3)	0.0473 (3)	0.0127 (2)	−0.0098 (3)	−0.0047 (2)
Cl3	0.0395 (3)	0.0437 (3)	0.0588 (3)	0.0141 (2)	0.0107 (2)	0.0032 (2)
Cl4	0.0436 (3)	0.0295 (2)	0.0461 (3)	−0.00512 (19)	−0.0052 (2)	0.0032 (2)
N1	0.0350 (8)	0.0266 (7)	0.0266 (7)	0.0031 (6)	−0.0034 (6)	0.0062 (6)
N2	0.0422 (9)	0.0291 (8)	0.0284 (8)	−0.0025 (7)	−0.0062 (7)	0.0064 (6)
N3	0.0512 (10)	0.0306 (8)	0.0330 (9)	0.0007 (7)	−0.0132 (7)	0.0041 (7)

### 5,5-Dichloro-2-(dichloromethyl)-6,6-dimethyl-5,6-dihydropyrimidin-4-amine Geometric parameters (Å, º)

**Table:**

C1—N2	1.276 (2)	C5—Cl1	1.760 (2)
C1—N1	1.387 (2)	C5—Cl2	1.774 (2)
C1—C5	1.504 (2)	C5—H5A	0.9800
C2—N2	1.473 (2)	C6—H6A	0.9600
C2—C7	1.526 (3)	C6—H6B	0.9600
C2—C6	1.536 (3)	C6—H6C	0.9600
C2—C3	1.541 (3)	C7—H7A	0.9600
C3—C4	1.525 (2)	C7—H7B	0.9600
C3—Cl4	1.7851 (18)	C7—H7C	0.9600
C3—Cl3	1.7960 (19)	N3—H3A	0.9000
C4—N1	1.307 (2)	N3—H3B	0.9001
C4—N3	1.313 (2)		
			
N2—C1—N1	129.26 (17)	C1—C5—H5A	108.5
N2—C1—C5	114.72 (16)	Cl1—C5—H5A	108.5
N1—C1—C5	115.98 (15)	Cl2—C5—H5A	108.5
N2—C2—C7	107.95 (15)	C2—C6—H6A	109.5
N2—C2—C6	107.44 (16)	C2—C6—H6B	109.5
C7—C2—C6	111.29 (18)	H6A—C6—H6B	109.5
N2—C2—C3	108.66 (14)	C2—C6—H6C	109.5
C7—C2—C3	111.77 (17)	H6A—C6—H6C	109.5
C6—C2—C3	109.58 (16)	H6B—C6—H6C	109.5
C4—C3—C2	108.96 (15)	C2—C7—H7A	109.5
C4—C3—Cl4	112.61 (12)	C2—C7—H7B	109.5
C2—C3—Cl4	111.15 (13)	H7A—C7—H7B	109.5
C4—C3—Cl3	105.30 (12)	C2—C7—H7C	109.5
C2—C3—Cl3	111.34 (12)	H7A—C7—H7C	109.5
Cl4—C3—Cl3	107.34 (10)	H7B—C7—H7C	109.5
N1—C4—N3	121.44 (17)	C4—N1—C1	116.08 (15)
N1—C4—C3	118.94 (15)	C1—N2—C2	116.41 (15)
N3—C4—C3	119.58 (16)	C4—N3—H3A	121.1
C1—C5—Cl1	110.83 (14)	C4—N3—H3B	122.3
C1—C5—Cl2	110.93 (14)	H3A—N3—H3B	116.6
Cl1—C5—Cl2	109.46 (11)		
			
N2—C2—C3—C4	−51.33 (19)	Cl3—C3—C4—N3	98.37 (18)
C7—C2—C3—C4	−170.35 (16)	N2—C1—C5—Cl1	117.03 (17)
C6—C2—C3—C4	65.79 (19)	N1—C1—C5—Cl1	−64.87 (19)
N2—C2—C3—Cl4	−176.00 (12)	N2—C1—C5—Cl2	−121.15 (17)
C7—C2—C3—Cl4	64.98 (19)	N1—C1—C5—Cl2	56.9 (2)
C6—C2—C3—Cl4	−58.88 (18)	N3—C4—N1—C1	175.14 (18)
N2—C2—C3—Cl3	64.38 (17)	C3—C4—N1—C1	−7.3 (2)
C7—C2—C3—Cl3	−54.64 (19)	N2—C1—N1—C4	−16.6 (3)
C6—C2—C3—Cl3	−178.50 (13)	C5—C1—N1—C4	165.66 (17)
C2—C3—C4—N1	40.3 (2)	N1—C1—N2—C2	1.1 (3)
Cl4—C3—C4—N1	164.11 (14)	C5—C1—N2—C2	178.85 (16)
Cl3—C3—C4—N1	−79.24 (18)	C7—C2—N2—C1	155.08 (19)
C2—C3—C4—N3	−142.10 (18)	C6—C2—N2—C1	−84.8 (2)
Cl4—C3—C4—N3	−18.3 (2)	C3—C2—N2—C1	33.7 (2)

### 5,5-Dichloro-2-(dichloromethyl)-6,6-dimethyl-5,6-dihydropyrimidin-4-amine Hydrogen-bond geometry (Å, º)

**Table:**

*D*—H···*A*	*D*—H	H···*A*	*D*···*A*	*D*—H···*A*
N3—H3*A*···N1^i^	0.90	2.08	2.978 (2)	174
N3—H3*B*···Cl4	0.90	2.53	2.9364 (17)	108
C5—H5*A*···N2^ii^	0.98	2.54	3.506 (3)	170
C6—H6*B*···Cl4	0.96	2.75	3.109 (2)	103
C7—H7*B*···Cl3	0.96	2.76	3.112 (2)	103
C7—H7*C*···Cl4	0.96	2.77	3.218 (2)	110

Symmetry codes: (i) −*x*, −*y*+1, −*z*+1; (ii) −*x*+1, −*y*+1, −*z*+2.

### Short interatomic contacts (Å)

**Table:**

Contact	Distance	Symmetry operation
Cl1···H6C	3.15	1+x,y,z
Cl2···H7C	3.07	x,1+y,z
H3A···N1	2.08	-x,1-y,1-z
H6A···Cl2	3.08	-x,1-y,2-z
H5A···N2	2.54	1-x,1-y,2-z

## References

[bb1] Aliyeva, V. A., Gurbanov, A. V., Huseynov, F. E., Hajiyeva, S. R., Conceição, N. R., Nunes, A. V. M., Pombeiro, A. J. L. & Mahmudov, K. T. (2024). *Polyhedron* **255**, 116955.

[bb2] Bernstein, J., Davis, R. E., Shimoni, L. & Chang, N.-L. (1995). *Angew. Chem. Int. Ed. Engl.* **34**, 1555–1573.

[bb3] Bruker (2008). *APEX3* and *SAINT*. Bruker AXS Inc., Madison, Wisconsin, USA.

[bb5] Farrugia, L. J. (2012). *J. Appl. Cryst.* **45**, 849–854.

[bb6] Gadzhieva, S. R., Guseinov, F. E. & Chyragov, F. M. (2005). *J. Anal. Chem.* **60**, 819–821.

[bb7] Groom, C. R., Bruno, I. J., Lightfoot, M. P. & Ward, S. C. (2016). *Acta Cryst.* B**72**, 171–179. 10.1107/S2052520616003954 PMC4822653 27048719

[bb8] Gurbanov, A. V., Gomila, R. M., Frontera, A., Shikhaliyev, N. Q., Zeynalli, N. R., Mahmudov, K. T. & Pombeiro, A. J. L. (2023). *Cryst. Growth Des.* **23**, 7647–7652.

[bb9] Gurbanov, A. V., Huseynov, F. E., Mahmoudi, G., Maharramov, A. M., Guedes da Silva, F. C., Mahmudov, K. T. & Pombeiro, A. J. L. (2018). *Inorg. Chim. Acta* **469**, 197–201.

[bb10] Gurbanov, A. V., Kuznetsov, M. L., Resnati, G., Mahmudov, K. T. & Pombeiro, A. J. L. (2022). *Cryst. Growth Des.* **22**, 3932–3940.

[bb11] Huseynov, F. E., Shamilov, N. T., Mahmudov, K. T., Maharramov, A. M., Guedes da Silva, M. F. C. & Pombeiro, A. J. L. (2018). *J. Organomet. Chem.* **867**, 102–105.

[bb12] Krause, L., Herbst-Irmer, R., Sheldrick, G. M. & Stalke, D. (2015). *J. Appl. Cryst.* **48**, 3–10. 10.1107/S1600576714022985 PMC4453166 26089746

[bb13] Maharramov, A. M., Gadzhieva, S. R., Bahmanova, F. N., Gamidov, S. Z. & Chyragov, F. M. (2011). *J. Anal. Chem.* **66**, 480–483.

[bb17] Mahmudov, K. T. & Pombeiro, A. J. L. (2023). *Chem. A Eur. J.* **29**, e202203861. 10.1002/chem.202203861 36815600

[bb18] Mori, Y. & Maeda, K. (1994). *Bull. Chem. Soc. Jpn* **67**, 1204–1206.

[bb19] Polyanskii, K. B., Alekseeva, K. A., Raspertov, P. V., Kumandin, P. A., Nikitina, E. V., Gurbanov, A. V. & Zubkov, F. I. (2019). *Beilstein J. Org. Chem.* **15**, 769–779. 10.3762/bjoc.15.73 PMC6444410 30992725

[bb20] Sheldrick, G. M. (2008). *Acta Cryst.* A**64**, 112–122. 10.1107/S0108767307043930 18156677

[bb21] Sheldrick, G. M. (2015). *Acta Cryst.* C**71**, 3–8.

[bb22] Spackman, P. R., Turner, M. J., McKinnon, J. J., Wolff, S. K., Grimwood, D. J., Jayatilaka, D. & Spackman, M. A. (2021). *J. Appl. Cryst.* **54**, 1006–1011. 10.1107/S1600576721002910 PMC8202033 34188619

[bb23] Spek, A. L. (2020). *Acta Cryst.* E**76**, 1–11. 10.1107/S2056989019016244 PMC6944088 31921444

[bb24] Wan, Q., Hou, Z. W., Zhao, Q., Xie, X. & Wang, L. (2023). *Org. Lett.* **25**, 1008–1013. 10.1021/acs.orglett.3c00144 36735345

